# Diabetes in a Young Child

**Published:** 1897-03

**Authors:** 


### DIABETES IN A YOUNG CHILD.

The chief interest in the following case, reported by Mr. Horace Wilson, of Port St. Mary, lies in the extreme youth of the patient. It exemplifies the advisability of examining the urine in all cases of nocturnal incontinence in private practice where this examination is not a proceeding of routine as in hospital work. Mr. Wilson was called to a child aged four years, the son of healthy parents, and the fourth child of a family of six, all of whom were strong and well with the one exception. The patient seemed to be well-nourished, bright and intelligent, and made no complaint of feeling badly. His mother, however, complained that he was growing thin and that he had been wetting the bed during the previous two weeks. On examination the bladder was found to be empty and the prepuce slightly excoriated, there was no tendency to phimosis, no pain or tenderness anywhere; the tongue was moist and of good color and the bowels regular. The tincture of belladonna was prescribed in small doses, and directions given to wash the prepuce twice daily with boracic lotion. A week afterwards the child was much paler, appeared more wasted, and was with difficulty aroused to answer questions. He had been vomiting constantly since the previous afternoon and had for the first time wetted his trousers, being apparently unaware of the fact. Close questioning of the parents elicited the facts that he had drank large quantities of water for the five weeks previously, and that, "although he had not been well, his appetite seemed to improve." On examining the urine the specific gravity was found to be 1035 and it contained six grains of sugar to the ounce. The total amount passed could not be estimated. On the following day he was pale and emaciated, with cold extremities and small pulse. His pupils reacted to light, but he was quite comatose; his breathing was heavy and labored, and he was making no effort to swallow. The bed beneath him was "floury" with the crystals. He never regained consciousness and succumbed two hours after the physician's last visit.—*Lancet.*

